# Perspectives in clinical research on Azathioprine for steroid-dependent ulcerative colitis

**DOI:** 10.3389/fmed.2025.1551906

**Published:** 2025-03-25

**Authors:** Yuan-Ting Qu, Jia-Yuan Ding, Wei Pan, Fang-Rui Liu, Ai-Lian Dong

**Affiliations:** ^1^Department of Gastroenterology, Hongqi Hospital Affiliated to Mudanjiang Medical University, Mudanjiang, China; ^2^Department of Gastroenterology, Mudanjiang First People’s Hospital, Mudanjiang, China

**Keywords:** ulcerative colitis, Azathioprine, inflammatory bowel disease, biomarkers, perspectives

## Abstract

This study explores the application of Azathioprine in the treatment of ulcerative colitis (UC) and the challenges associated with its long-term use. While short-term studies demonstrate the efficacy of Azathioprine in steroid-dependent UC, long-term data on its risks, including malignancies, infections, and chronic toxicity, remain insufficient. Furthermore, the impact of Azathioprine on patients’ quality of life over extended periods is still unclear. The research highlights the importance of optimizing Azathioprine dosing based on genomic data, particularly through TPMT and NUDT15 genotyping, to minimize adverse effects. However, further research is needed to develop individualized treatment strategies that can improve efficacy and reduce toxicity. The identification of predictive biomarkers, through genomics and proteomics, is likely to play a crucial role in improving treatment precision by identifying patients who are most likely to benefit from Azathioprine therapy. Additionally, combining Azathioprine with biologic therapies (such as anti-TNF agents or integrin inhibitors) and interventions targeting the gut microbiome may enhance the drug’s effectiveness while reducing reliance on steroids. Overall, large-scale clinical trials are urgently needed to evaluate the benefits and risks of these emerging therapies, ultimately supporting more personalized treatment approaches for steroid-dependent UC patients.

## 1 Introduction

Ulcerative colitis (UC) is a chronic, relapsing inflammatory bowel disease characterized by inflammation and ulceration of the colonic mucosa, typically involving the rectum and colon (1). The disease follows a variable course, with periods of remission and flare-ups. UC has a global prevalence, with higher rates observed in developed countries, especially in North America and Europe (2). It is most commonly diagnosed in young adults, particularly between the ages of 20 and 40 years, although it can affect individuals at any age (3).

The pathogenesis of UC is multifactorial, involving a complex interaction between genetic susceptibility, environmental factors, and immune system dysregulation (4). In steroid-dependent UC, patients experience chronic inflammation and mucosal injury that require prolonged corticosteroid use to manage flare-ups (5). However, this long-term corticosteroid dependency is accompanied by significant concerns, including the development of side effects such as osteoporosis, hypertension, and glucose intolerance (6).

Corticosteroids have long been the mainstay of treatment for UC flare-ups due to their potent anti-inflammatory properties. However, their prolonged use is associated with numerous adverse effects, such as weight gain, bone demineralization, increased infection risk, and metabolic disturbances, including glucose intolerance and hypertension (7, 8). These risks are particularly problematic in patients with steroid-dependent UC, as they require continuous corticosteroid therapy to manage their symptoms, leading to a cycle of dependency (7, 8). This situation underscores the pressing need for alternative, steroid-sparing therapies that can provide effective disease control while minimizing the risks of corticosteroid-related complications (8). In recent years, various immunosuppressive therapies, biologic agents, and small-molecule drugs have been developed to address this need, offering promising options for achieving remission and improving the overall quality of life for patients (9).

Azathioprine, an immunosuppressive agent, has emerged as a key treatment for steroid-dependent UC (10). As a purine analog, Azathioprine works by inhibiting purine synthesis, disrupting DNA and RNA production in rapidly proliferating immune cells, particularly T and B lymphocytes (10, 11). This results in a reduction in the inflammatory immune response that drives UC pathology. Azathioprine is metabolized into its active form, 6-mercaptopurine (6-MP), which is responsible for its therapeutic effects (12).

Initially introduced as a second-line therapy for patients who were unresponsive to or intolerant of corticosteroids, Azathioprine is now primarily used for maintaining remission in steroid-dependent UC, rather than for inducing remission (12). Numerous clinical studies have shown that Azathioprine effectively reduces steroid dependency, controls flare-ups, and improves long-term disease management (11, 13–22). However, its use requires careful monitoring due to potential side effects, such as bone marrow suppression, hepatotoxicity, and an increased risk of infections (14–17, 20). Despite these risks, Azathioprine remains a critical tool in the management of steroid-dependent UC, providing an effective steroid-sparing alternative for patients seeking long-term remission and improved disease control.

## 2 Study search and selection

A systematic search strategy was devised to identify studies evaluating the use of Azathioprine in the treatment of steroid-dependent UC. The search was conducted across two major databases: PubMed and China National Knowledge Infrastructure, employing key search terms including “Azathioprine,” “ulcerative colitis,” and “steroid-dependent.” This approach aimed to ensure a broad representation of studies from both international and local sources. No restrictions were applied based on publication date, and studies published in English and Chinese were included to encompass the global body of research available up to October 1, 2024.

To be eligible for inclusion, studies had to meet the following criteria: (1) they specifically focused on Azathioprine as a treatment for steroid-dependent UC; (2) they employed one of the following study designs: randomized controlled trials, retrospective studies, or observational studies; and (3) they were published in English or Chinese. Studies were excluded if they did not meet these criteria, including those that did not specifically address Azathioprine in the context of steroid-dependent UC, duplicates, or studies with non-randomized designs such as cohort studies, case reports, or systematic reviews without original data.

**Figure 1:**
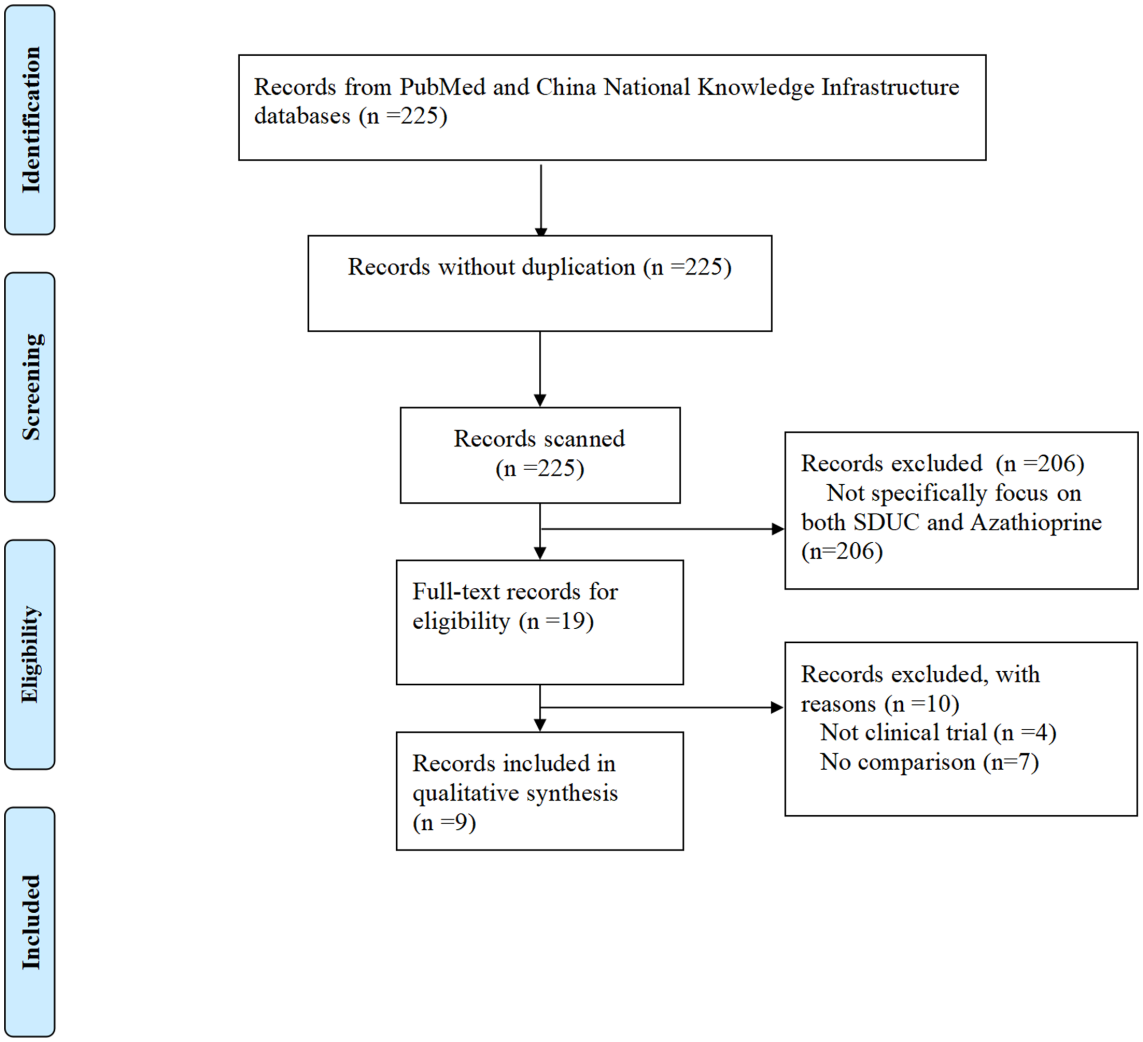
Flowchart of study selection.

A total of 225 studies were identified initially. After applying the eligibility criteria, 206 studies were excluded for being irrelevant to the topic or failing to meet the inclusion criteria. The remaining 19 studies were subject to full-text review. Of these, 10 studies were excluded due to the following reasons: they were not clinical trials or lacked a comparison group. Ultimately, 9 studies met the eligibility criteria and were included in the final analysis (Figure 1).

## 3 Mechanism of action and pharmacology of Azathioprine

### 3.1 Overview of Azathioprine’s mechanism

Azathioprine is a purine analog with immunosuppressive properties, primarily exerted through the inhibition of purine synthesis (10). This inhibition disrupts DNA and RNA production in proliferating cells, which are especially critical in the immune response. Once ingested, Azathioprine is metabolized into its active form, 6-MP, in the liver. 6-MP inhibits the enzyme inosine monophosphate dehydrogenase (IMPDH), which plays a key role in the *de novo* purine synthesis pathway (10–12). By reducing the availability of purine nucleotides, Azathioprine limits the proliferation of immune cells such as T-cells and B-cells, which are central to the pathogenesis of UC (11, 23). This suppression of immune cells reduces the excessive inflammatory response that drives the disease. Additionally, Azathioprine modulates several inflammatory pathways in UC, notably through the reduction of pro-inflammatory cytokines like tumor necrosis factor-alpha (TNF-*α*), interleukin-1 beta, and interleukin-6 (18, 22). This multifaceted mechanism contributes to the reduction in colonic inflammation and helps control disease activity, providing a steroid-sparing alternative for patients with steroid-dependent UC.

### 3.2 Pharmacokinetics and pharmacodynamics

The pharmacokinetics of Azathioprine are crucial in determining its therapeutic efficacy and safety profile (10). Following oral administration, Azathioprine is well absorbed, with peak plasma concentrations reached within 1–2 h. However, its bioavailability can vary, with estimates suggesting it is around 50% (24). Once absorbed, Azathioprine is rapidly converted into its active metabolite, 6-MP, primarily in the liver (25). 6-MP is further metabolized into both inactive metabolites and its active form, which exerts the therapeutic effects. One key metabolic pathway involves the enzyme thiopurine methyltransferase (TPMT), which is responsible for converting 6-MP into its inactive metabolites (25). The activity of TPMT is genetically determined, and genetic polymorphisms can significantly influence both the efficacy and toxicity of Azathioprine (25). Patients with reduced TPMT activity metabolize Azathioprine more slowly, leading to higher concentrations of 6-MP, which can increase the risk of adverse effects (25). Conversely, individuals with high TPMT activity may metabolize the drug more rapidly, potentially reducing its efficacy (24). In addition to TPMT, recent studies have highlighted the importance of another genetic variant, NUDT15, in predicting Azathioprine-induced leukopenia and optimizing dosing strategies (26, 27). NUDT15 is an enzyme involved in the metabolism of thiopurine drugs, and mutations in the NUDT15 gene, particularly the C415T variant, have been associated with an increased risk of severe myelosuppression in patients receiving Azathioprine (28). Notably, NUDT15 mutations are more prevalent in Asian populations, and genotyping for NUDT15 is recommended alongside TPMT testing to personalize Azathioprine dosing and minimize toxicity (27). Therefore, pre-treatment testing for both TPMT and NUDT15 polymorphisms is critical to ensure safe and effective use of Azathioprine in patients with steroid-dependent UC.

### 3.3 Dose–response and safety considerations

The dosing of Azathioprine in steroid-dependent UC requires careful individualization to balance efficacy and safety (11). The usual starting dose is between 1.5 to 2.5 mg/kg body weight per day, depending on the patient’s clinical condition and response to the drug. Adjustments may be made based on therapeutic goals, side effects, and patient tolerance (11). Monitoring during treatment is essential to ensure the drug is both effective and well-tolerated (29). Key parameters to assess include complete blood counts to detect leukopenia or thrombocytopenia, liver function tests to monitor hepatotoxicity, and signs of infection (29, 30). Leukopenia, a reduction in white blood cells, is a common side effect of Azathioprine and increases the risk of infections, making regular monitoring crucial (29). Hepatotoxicity, often indicated by elevated liver enzymes, is another serious adverse effect that requires attention (30).

## 4 Clinical efficacy of Azathioprine in steroid-dependent UC

### 4.1 Summary of clinical trials

Azathioprine has been extensively studied for its efficacy in managing steroid-dependent UC, particularly its ability to reduce corticosteroid dependence and maintain sustained remission (14–22) (Table 1). A pivotal randomized controlled trial by Ardizzone et al. demonstrated that Azathioprine achieved clinical and endoscopic remission in 53% of patients compared to 21% in the 5-aminosalicylic acid group, emphasizing its superior efficacy in steroid tapering and remission maintenance (14). However, it is important to note that current guidelines, such as those from the European Crohn’s and Colitis Organization and the American Gastroenterological Association, recommend against using Azathioprine as an induction therapy due to its slow onset of action and the availability of more effective induction agents, such as biologics and corticosteroids (31, 32). Instead, Azathioprine is best utilized as a maintenance therapy to sustain remission and reduce steroid dependency. Similarly, Mantzaris et al. highlighted the comparable efficacy of Azathioprine monotherapy and its combination with olsalazine, affirming Azathioprine as a viable standalone option for maintaining remission in steroid-dependent UC (15).

**Table 1:** Summary of included studies.

Study	Patient information	Sample size	Publication type	Treatment	Main findings
Ardizzone et al., 2006 (14)	Patients with SDUC	72	RCT	AZA or 5-aminosalicylic acid	AZA outperforms 5-aminosalicylic acid in inducing remission and reducing steroid use in SDUC.
Mantzaris et al., 2004 (15)	Patients with SDUC	70	RCT	AZA alone or with olsalazine	Patients with SDUC in remission on AZA do not require 5-aminosalicylic acid compounds.
Zheng et al., 2019 (16)	Patients with SDUC	80	RCT	Glucocorticoids alone or with AZA	AZA is effective and safe for SDUC.
Lin et al., 2017 (17)	Patients with SDUC	40	RCT	Glucocorticoids alone or with AZA	AZA effectively replaces corticosteroids in SDUC, reducing corticosteroid use and minimizing side effects.
Chen et al., 2016 (18)	Patients with SDUC	90	RCT	Glucocorticoids alone, with AZA, or with both AZA and MHUD	AZA combined with MHUD supports immune function, reduces inflammation, and promotes colonic and liver health in SDUC patients.
Park et al., 2013 (19)	Patients with SDUC	106	Retrospective study	AZA	AZA is an effective and long-lasting treatment for SDUC, with sustained efficacy over 3 years.
Ardizzone et al., 1997 (20)	Patients with SDUC	56	Retrospective study	AZA	AZA effectively prevents colectomy in steroid-resistant and SDUC, reducing steroid use and clinical relapses.
Wang et al., 2017 (21)	Patients with SDUC	75	Observational study	AZA or prednisone	AZA outperforms steroids in treating SDUC and is safe.
Lin et al., 2017 (22)	Patients with SDUC	40	Observational study	AZA or prednisone	AZA enhances efficacy and lowers IL-8 and TNF-*α* in SDUC.

SDUC, steroid-dependent ulcerative colitis; RCT, randomized controlled trial; AZA, Azathioprine; MDUD, Modified Healing Ulcer Decoction.

In long-term assessments, Park et al. reported a remission rate of 54.7% over three years, with the success rate increasing to 71.2% among patients who continued Azathioprine therapy beyond six months. These findings underscore its enduring role in UC management (19). Furthermore, retrospective studies, such as those by Zheng and Lin et al., corroborate Azathioprine’s effectiveness, albeit with inherent limitations due to study design and cohort size (16, 17). Collectively, these trials highlight Azathioprine’s potential as a cost-effective and accessible therapy, particularly in settings where biologics may not be readily available (Table 1).

### 4.2 Critical analysis of clinical trials

While the existing clinical trials demonstrate the efficacy of Azathioprine in steroid-dependent UC, several limitations should be acknowledged. Many studies, such as those by Lin et al., suffer from small sample sizes, short follow-up periods, which restrict the generalizability of their findings (17, 22). For instance, the study by Lin et al. included only 40 patients with a follow-up period of six months, which may not adequately capture long-term outcomes or rare adverse events (22). Additionally, the heterogeneity of UC severity across studies complicates the interpretation of results, as patients with extensive or refractory disease may exhibit suboptimal responses to Azathioprine monotherapy. Moreover, the lack of standardized dosing protocols and monitoring strategies across studies further limits the comparability of results. For example, some studies initiated Azathioprine at a fixed dose (14–18). These methodological shortcomings emphasize the need for large-scale, long-term, randomized controlled trials with standardized protocols to refine treatment guidelines and provide more robust evidence for Azathioprine’s use in diverse UC populations.

### 4.3 Efficacy in inducing and maintaining remission

Azathioprine is highly effective in maintaining remission in steroid-dependent UC, but its role in inducing remission is limited due to its slow onset of action. While some studies have reported remission within two to four months of initiation, current guidelines recommend against using Azathioprine as an induction therapy, favoring faster-acting agents such as biologics or corticosteroids for this purpose (31, 32). Instead, Azathioprine is best utilized as a maintenance therapy to sustain remission and reduce steroid dependency (Table 1). For instance, Lin et al. observed that Azathioprine treatment not only decreased corticosteroid dependency but also reduced the occurrence of corticosteroid-related adverse effects, such as gastrointestinal ulcers and Cushing’s syndrome (17).

Its long-term utility is equally remarkable. Wang et al. reported that Azathioprine achieved a clinical efficacy rate of 92.1%, significantly outperforming corticosteroid therapy. Additionally, combination strategies have been explored to enhance Azathioprine’s effectiveness (21). Chen et al. demonstrated that combining Azathioprine with modified Yuanyang Decoction improved mucosal healing and significantly reduced inflammatory markers such as interleukin-8 and TNF-*α* compared to monotherapy (18, 22). Modified Yuanyang Decoction, a traditional Chinese medicine formulation, has been shown to possess anti-inflammatory and immunomodulatory properties, which may synergize with Azathioprine to enhance mucosal healing and reduce disease activity in steroid-dependent UC patients (18). Further studies are needed to elucidate the mechanisms underlying this synergistic effect and to optimize the dosing and administration of this combination therapy. Similarly, studies have suggested that Azathioprine, when combined with biologics or corticosteroids, can expedite disease control and prolong remission in severe or refractory UC cases (18, 19) (Table 1).

### 4.4 Challenges in efficacy assessment

Despite its clinical utility, challenges remain in evaluating Azathioprine’s full therapeutic potential. One significant issue is variability in patient response, often influenced by genetic factors. This variability underscores the necessity of pharmacogenetic testing prior to initiating Azathioprine therapy to optimize outcomes and minimize risks (19) (Table 1).

Moreover, the heterogeneity of UC severity impacts Azathioprine’s efficacy, with patients suffering from extensive or refractory disease often exhibiting suboptimal responses to monotherapy (19, 20) (Table 1). Another challenge lies in the limitations of existing clinical trials. Many studies, such as those by Zheng and Lin et al., suffer from small sample sizes, short follow-up periods, and retrospective designs, which restrict the generalizability of their findings (16, 17). These methodological shortcomings emphasize the need for large-scale, long-term, randomized controlled trials to refine treatment protocols and broaden the evidence base for Azathioprine’s use in diverse UC populations.

In summary, Azathioprine remains a cornerstone in the management of steroid-dependent UC due to its proven efficacy in reducing corticosteroid dependence, maintaining remission, and improving mucosal healing (Table 1). However, its clinical application is complicated by patient variability, disease severity, and limitations in current evidence. To maximize its therapeutic potential, future research should focus on addressing these challenges through pharmacogenetic approaches, combination strategies, and robust clinical trials. By doing so, Azathioprine’s role in personalized UC management can be further enhanced, offering improved outcomes for a broader range of patients.

## 5 Safety and side effects of Azathioprine in UC patients

Azathioprine, while effective in treating steroid-dependent UC, is associated with a range of adverse effects (14–17, 20). Common side effects include gastrointestinal disturbances, such as nausea, vomiting, and diarrhea, which are often dose-dependent (29). Bone marrow suppression, including leukopenia, thrombocytopenia, and anemia, is a more serious concern, leading to increased susceptibility to infections (30, 33). Although rare, severe adverse events include an elevated risk of malignancies, particularly lymphomas and skin cancers, as well as serious infections, such as opportunistic pathogens, due to the drug’s immunosuppressive properties (29). This underscores the need for vigilant patient monitoring during treatment.

Long-term safety concerns with Azathioprine use have been increasingly documented in recent studies. Yewale et al. conducted a real-world study evaluating the long-term safety and effectiveness of Azathioprine in inflammatory bowel disease (IBD) patients (34). Their findings highlighted that while Azathioprine is effective in maintaining remission, long-term use is associated with an increased risk of malignancies, particularly lymphoproliferative disorders and non-melanoma skin cancers (34, 35). However, the reported rate of malignancy was relatively low (1.8%), and these results were corroborated by a recent longitudinal follow-up study by Ranjan et al., which also emphasized a minimal risk of lymphoma and non-melanoma skin cancer despite long-term use of thiopurines in IBD patients (34, 36). The conclusion of both studies supports the long-term use of Azathioprine, although larger, more comprehensive studies are needed to further address its long-term safety profile in IBD. Additionally, Singh et al. provided an updated review on the use of thiopurines in IBD, emphasizing that prolonged Azathioprine therapy is linked to an increased incidence of intolerance or adverse effects (35). These studies underscore the importance of regular surveillance for malignancies and infections in patients on long-term Azathioprine therapy.

To minimize risks, regular monitoring is essential. Pre-treatment testing for genetic variations is critical to minimize the risk of severe bone marrow suppression (37). Routine blood tests to monitor white blood cell counts, liver function, and kidney function are essential throughout treatment (29). Monitoring should be more frequent during the initial stages, with adjustments based on the patient’s response to therapy. Liver function tests are particularly important due to the risk of hepatotoxicity associated with Azathioprine (30).

Long-term use of Azathioprine raises additional safety concerns, particularly regarding its impact on quality of life. Persistent gastrointestinal symptoms and a heightened risk of malignancy can reduce patient well-being (30, 38). The drug’s immunosuppressive effects may also exacerbate comorbid conditions such as osteoporosis and cardiovascular disease, which are common in UC patients (39, 40). Given these potential risks, regular surveillance for malignancies, particularly skin cancer, is recommended. Clinicians should also be vigilant about managing comorbid conditions to mitigate the broader health impacts of prolonged Azathioprine use (41). These long-term considerations highlight the need for careful risk–benefit assessment, especially for patients requiring extended treatment to maintain remission.

## 6 Azathioprine resistance and non-responders

### 6.1 Mechanisms of resistance

Azathioprine, despite its efficacy in treating steroid-dependent UC, may fail in some patients due to a variety of resistance mechanisms, which can be classified into genetic and environmental factors (37). Genetic variations play a crucial role in determining the response to Azathioprine (37). Notably, polymorphisms in drug-metabolizing enzymes, such as NUDT15, can significantly impact drug metabolism, leading to suboptimal therapeutic outcomes or severe adverse events (37). NUDT15 mutations, particularly the C415T variant, have been associated with an increased risk of Azathioprine-induced leukopenia, especially in Asian populations (26, 27). These genetic variations underscore the importance of pharmacogenetic testing prior to initiating Azathioprine therapy to optimize dosing and minimize the risk of toxicity (28). In addition, genetic variations in other enzymes such as IMPDH, involved in purine metabolism, may also influence the drug’s effectiveness (42).

Environmental factors, including gut microbiota composition, co-existing infections, or concurrent inflammatory stimuli, may exacerbate resistance (43). Alterations in the microbiome can influence the immune response, reducing the efficacy of immunosuppressive therapies like Azathioprine (43). Additionally, chronic inflammation in UC patients may upregulate pro-inflammatory cytokines, potentially bypassing the effects of Azathioprine and contributing to continued disease activity despite treatment (44).

### 6.2 Strategies for managing non-responders

Managing patients who do not respond to Azathioprine requires a multifaceted approach, incorporating therapeutic drug monitoring (TDM), dose optimization, and combination therapies (45). TDM is a critical tool in optimizing Azathioprine therapy. TDM involves measuring the levels of active metabolites, such as 6-thioguanine nucleotides (6-TGN), to ensure that patients are within the therapeutic range. Subtherapeutic levels of 6-TGN may result in treatment failure, while excessive levels can lead to toxicity. Regular monitoring of 6-TGN levels allows clinicians to adjust the dose based on the patient’s metabolic profile, thereby improving efficacy and minimizing adverse effects (46).

In cases where TDM indicates suboptimal drug levels, dose escalation may be considered, provided that the patient does not exhibit signs of toxicity. However, dose adjustments should be guided by genetic testing for TPMT and NUDT15 polymorphisms, as these variants significantly influence drug metabolism and toxicity risk (37, 47, 48).

For patients who remain unresponsive despite optimal dosing, alternative treatment strategies should be explored. Combination therapies represent another effective approach for managing non-responders. Combining Azathioprine with biologics such as anti-TNF agents (e.g., infliximab) or integrin inhibitors (e.g., vedolizumab) can enhance anti-inflammatory effects and improve disease control (46). Studies have shown that combination therapy with Azathioprine and biologics can lead to higher rates of mucosal healing and prolonged remission compared to monotherapy (46). Additionally, the use of JAK inhibitors (e.g., tofacitinib) in combination with Azathioprine has shown promise in refractory cases, particularly in patients with severe or extensive disease (49).

For patients who fail to respond to combination therapies, switching to biologic monotherapy may be necessary. Biologics such as anti-TNF agents, integrin inhibitors, or interleukin inhibitors (e.g., ustekinumab) have demonstrated efficacy in inducing and maintaining remission in patients with steroid-dependent UC (50, 51). The choice of biologic should be guided by the patient’s disease severity, prior treatment history, and potential side effects.

## 7 Current research and future directions

### 7.1 Emerging therapeutic approaches

Recent advancements in UC treatment have introduced novel therapies aimed at enhancing the efficacy of Azathioprine, particularly in steroid-dependent cases. The combination of immunosuppressive agents such as anti-TNF (e.g., infliximab) and integrin inhibitors (e.g., vedolizumab) with Azathioprine is increasingly explored for its potential to improve disease control and reduce reliance on corticosteroids (46). These biologics target specific immune pathways involved in UC, offering a complementary approach to traditional immunosuppressants.

In addition to biologics, JAK inhibitors (e.g., tofacitinib, upadacitinib) have emerged as a promising class of drugs for the treatment of UC. JAK inhibitors target the Janus kinase-signal transducer and activator of transcription (JAK–STAT) pathway, which plays a key role in the pathogenesis of UC by regulating the production of pro-inflammatory cytokines (49). Recent studies have suggested that combining Azathioprine with JAK inhibitors may enhance therapeutic efficacy, particularly in patients with refractory or severe UC (52). This combination approach may allow for lower doses of each drug, potentially reducing the risk of adverse effects while maintaining disease control. However, further research is needed to evaluate the safety and efficacy of this combination, particularly in the context of long-term use.

In addition to its role in maintaining remission, Azathioprine has been widely reported to prevent the development of auto-antibodies and immunogenicity against anti-TNF agents, particularly infliximab (53). This combination therapy can enhance the efficacy of biologics by reducing the risk of anti-drug antibody formation, thereby improving long-term disease control (54, 55).

Moreover, the modulation of the gut microbiome is gaining traction as a therapeutic strategy. Techniques such as fecal microbiota transplantation or the use of probiotics aim to restore microbial balance in the gut, potentially enhancing the effectiveness of Azathioprine and reducing inflammation, which could be pivotal in managing steroid dependency (56).

### 7.2 Personalized medicine and Azathioprine

The future of UC treatment lies in personalized medicine, where therapies are tailored to the individual patient’s genetic profile. Genomic research focusing on TPMT and NUDT15 polymorphisms has significantly influenced the clinical application of Azathioprine, guiding dosage adjustments to minimize toxicity and improve efficacy (57). Recent studies have demonstrated that NUDT15 genotyping, particularly in Asian populations, can help predict the risk of Azathioprine-induced leukopenia and optimize treatment strategies (26, 27). Additionally, proteomic approaches are being explored to identify biomarkers that could predict a patient’s response to treatment, allowing for more precise and targeted therapy (58). Monitoring inflammatory markers such as C-reactive protein and fecal calprotectin can provide valuable insights into ongoing disease activity and predict the likelihood of response to therapy (59). The integration of pharmacogenomic testing and biomarker monitoring into clinical practice holds promise for improving outcomes by optimizing Azathioprine dosing and reducing adverse effects. Future research should focus on identifying additional predictive biomarkers, such as inflammatory markers (e.g., C-reactive protein and fecal calprotectin), to further refine personalized treatment strategies (59). This approach will help minimize the trial-and-error process typically associated with immunosuppressive therapies and improve the overall efficacy of Azathioprine in steroid-dependent UC patients.

### 7.3 Challenges and unanswered questions

Despite these advancements, there remain significant knowledge gaps in the long-term efficacy and safety of Azathioprine. While short-term studies show promising results, long-term data on the risk of malignancies, infections, and chronic toxicity are limited (60). Furthermore, the impact on quality of life over extended periods of use remains unclear. To address these concerns, larger, more comprehensive clinical trials are needed to assess the sustained benefits and risks of Azathioprine therapy in UC patients (61). Additionally, future research should focus on discovering predictive biomarkers to help identify patients who may benefit most from Azathioprine and other adjunctive therapies, such as biologics or microbiome interventions, ultimately improving treatment efficacy and minimizing adverse outcomes (62).

## 8 Summary

Azathioprine remains a key treatment for steroid-dependent UC, primarily effective in maintaining remission rather than inducing it. Its immunosuppressive properties help reduce inflammation, but its slow onset of action limits its use as an induction therapy. Current clinical guidelines endorse Azathioprine as a maintenance therapy to prolong remission and minimize steroid dependency. However, its therapeutic efficacy and safety profile are significantly influenced by genetic factors, particularly polymorphisms in the TPMT and NUDT15 genes, which affect drug metabolism and increase the risk of adverse events such as bone marrow suppression and hepatotoxicity. Long-term use of Azathioprine is associated with significant safety concerns, including an increased risk of malignancies and infections. To optimize treatment outcomes, personalized therapeutic strategies are essential, including genetic testing for TPMT and NUDT15 polymorphisms to guide dosing and mitigate risks. Furthermore, combination therapies, especially with biologics, may offer benefits for patients with inadequate responses to monotherapy. Rigorous and regular monitoring for potential side effects is imperative to ensure the safe and effective use of Azathioprine in clinical practice. Moving forward, integrating genomic biomarkers, novel biologics, and microbiome-based therapies may further optimize treatment strategies, enhancing both efficacy and safety for steroid-dependent UC patients.

## Data Availability

The original contributions presented in the study are included in the article/supplementary material, further inquiries can be directed to the corresponding author.

## References

[1] Gros B Kaplan GG. Ulcerative colitis in adults: a review. JAMA. (2023) 330:951–65. doi: 10.1001/jama.2023.15389, PMID: 37698559

[2] da Silva BC Lyra AC Rocha R Santana GO. Epidemiology, demographic characteristics and prognostic predictors of ulcerative colitis. World J Gastroenterol. (2014) 20:9458–67. doi: 10.3748/wjg.v20.i28.9458, PMID: 25071340 PMC4110577

[3] Duricova D Burisch J Jess T Gower-Rousseau C Lakatos PL ECCO-EpiCom. Age-related differences in presentation and course of inflammatory bowel disease: an update on the population-based literature. J Crohns Colitis. (2014) 8:1351–61. doi: 10.1016/j.crohns.2014.05.006, PMID: 24951261

[4] Ungaro R Mehandru S Allen PB Peyrin-Biroulet L Colombel JF. Ulcerative colitis. Lancet. (2017) 389:1756–70. doi: 10.1016/S0140-6736(16)32126-2, PMID: 27914657 PMC6487890

[5] Ramos GP Papadakis KA. Mechanisms of disease: inflammatory bowel diseases. Mayo Clin Proc. (2019) 94:155–65. doi: 10.1016/j.mayocp.2018.09.013 30611442 PMC6386158

[6] Mowat C Cole A Windsor A Ahmad T Arnott I Driscoll R. Guidelines for the management of inflammatory bowel disease in adults. Gut. (2011) 60:571–607. doi: 10.1136/gut.2010.224154, PMID: 21464096

[7] Lichtenstein GR Loftus EV Isaacs KL Regueiro MD Gerson LB Sands BE. ACG Clinical Guideline: Management of Crohn's disease in adults. Am J Gastroenterol. (2018) 113:481–517. doi: 10.1038/ajg.2018.27 29610508

[8] Harbord M Eliakim R Bettenworth D Karmiris K Katsanos K Kopylov U. Third European evidence-based consensus on diagnosis and Management of Ulcerative Colitis. Part 2: Current Management. J Crohns Colitis. (2017) 11:769–84. doi: 10.1093/ecco-jcc/jjx009, PMID: 28513805

[9] Lamb CA Kennedy NA Raine T Hendy PA Smith PJ Limdi JK. British Society of Gastroenterology consensus guidelines on the management of inflammatory bowel disease in adults. Gut. (2019) 68:s1–s106. doi: 10.1136/gutjnl-2019-318484, PMID: 31562236 PMC6872448

[10] Sasaki M Fujiyama Y. Azathioprine. Nihon Rinsho. (2005) 63:692–9. 15954431

[11] Timmer A Patton PH Chande N McDonald JW MacDonald JK. Azathioprine and 6-mercaptopurine for maintenance of remission in ulcerative colitis. Cochrane Database Syst Rev. (2016) 2016:CD000478. doi: 10.1002/14651858.CD000478.pub4, PMID: 27192092 PMC7034525

[12] Mallick B Malik S. Use of Azathioprine in ulcerative colitis: a comprehensive review. Cureus. (2022) 14:e24874. doi: 10.7759/cureus.24874, PMID: 35698683 PMC9184176

[13] Chebli LA Chaves LD Pimentel FF Guerra DM Barros RM Gaburri PD. Azathioprine maintains long-term steroid-free remission through 3 years in patients with steroid-dependent ulcerative colitis. Inflamm Bowel Dis. (2010) 16:613–9. doi: 10.1002/ibd.21083, PMID: 19705415

[14] Ardizzone S Maconi G Russo A Imbesi V Colombo E Bianchi PG. Randomised controlled trial of Azathioprine and 5-aminosalicylic acid for treatment of steroid dependent ulcerative colitis. Gut. (2006) 55:47–53. doi: 10.1136/gut.2005.068809, PMID: 15972298 PMC1856376

[15] Mantzaris GJ Sfakianakis M Archavlis E Petraki K Christidou A Karagiannidis A. A prospective randomized observer-blind 2-year trial of Azathioprine monotherapy versus Azathioprine and olsalazine for the maintenance of remission of steroid-dependent ulcerative colitis. Am J Gastroenterol. (2004) 99:1122–8. doi: 10.1111/j.1572-0241.2004.11481.x, PMID: 15180735

[16] Zheng M. Evaluation of the efficacy and safety of Azathioprine in the treatment of steroid-dependent ulcerative colitis. World Latest Med Inf Abstr. (2019) 19:104.

[17] Lin H Yao G Li Q Yang W Li H Huang G. Long-term efficacy observation of Azathioprine in the treatment of steroid-dependent ulcerative colitis patients. Intern Med. (2017) 12:672–4.

[18] Chen J Chen J Deng J Han Y. Clinical study on the combination of Azathioprine and modified Yuanyang decoction in steroid-dependent ulcerative colitis patients. Chin J Integr Dig Med. (2016) 24:287–9.

[19] Park SK Yang SK Ye BD Kim KJ Yang DH Jung KW. The long-term efficacy of Azathioprine in steroid-dependent ulcerative colitis. Scand J Gastroenterol. (2013) 48:1386–93. doi: 10.3109/00365521.2013.845908, PMID: 24164382

[20] Ardizzone S Molteni P Imbesi V Bollani S Bianchi PG. Azathioprine in steroid-resistant and steroid-dependent ulcerative colitis. J Clin Gastroenterol. (1997) 25:330–3. doi: 10.1097/00004836-199707000-00007, PMID: 9412914

[21] Wang H. Evaluation of the efficacy and safety of Azathioprine in the treatment of steroid-dependent ulcerative colitis. Chin J Min Med. (2017) 12:32–4. doi: 10.1186/s13020-017-0153-x, PMID: 29093747 PMC5663075

[22] Lin H Yao G Li Q Yang W Li H Huang G. Clinical study on the levels of IL-8 and TNF-α in the treatment of steroid-dependent ulcerative colitis with Azathioprine. Qiqihar Med J. (2017) 38:178–9.

[23] Fraser AG Orchard TR Jewell DP. The efficacy of Azathioprine for the treatment of inflammatory bowel disease: a 30 year review. Gut. (2002) 50:485–9. doi: 10.1136/gut.50.4.485, PMID: 11889067 PMC1773162

[24] Lennard L. The clinical pharmacology of 6-mercaptopurine. Eur J Clin Pharmacol. (1992) 43:329–39. doi: 10.1007/BF02220605, PMID: 1451710

[25] Derijks LJ Gilissen LP Engels LG Bos LP Bus PJ Lohman JJ. Pharmacokinetics of 6-mercaptopurine in patients with inflammatory bowel disease: implications for therapy. Ther Drug Monit. (2004) 26:311–8. doi: 10.1097/00007691-200406000-00016, PMID: 15167634

[26] Xu Y Qiao YQ Li HY Zhou M Cai CW Shen J. NUDT15 genotyping during Azathioprine treatment in patients with inflammatory bowel disease: implications for a dose-optimization strategy. Gastroenterol Rep. (2020) 8:437–44. doi: 10.1093/gastro/goaa021 PMC7793196 33442476

[27] Banerjee R Ravikanth VV Pal P Bale G Avanthi US Goren I. NUDT15 C415T variant compared with TPMT genotyping in predicting Azathioprine-induced leucopenia: prospective analysis of 1014 inflammatory bowel disease patients in India. Aliment Pharmacol Ther. (2020) 52:1683–94. doi: 10.1111/apt.16137 33111378

[28] Suzuki S Uchiyama K Motoi Y Yoshii Y Inoue Y Kubota T. Analysis of the NUDT15 gene and metabolites of Azathioprine in Japanese patients with inflammatory bowel disease. BMC Gastroenterol. (2023) 23:239. doi: 10.1186/s12876-023-02881-6 37454061 PMC10350251

[29] Reuther LO Sonne J Larsen NE Larsen B Christensen S Rasmussen SN. Pharmacological monitoring of Azathioprine therapy. Scand J Gastroenterol. (2003) 38:972–7. doi: 10.1080/00365520310005082, PMID: 14531535

[30] Bianchetti D Salvador Nunes L André P Schoepfer A Moradpour D Chtioui H. Metabolism and therapeutic monitoring of Azathioprine in gastroenterology and hepatology. Rev Med Suisse. (2022) 18:1588–93. doi: 10.53738/REVMED.2022.18.793.1588, PMID: 36047549

[31] Sriranganathan D Segal JP Garg M. Biologics recommendations in the ECCO guidelines on therapeutics in Crohn's disease: medical treatment. Frontline Gastroenterol. (2021) 13:168–70. doi: 10.1136/flgastro-2021-101881, PMID: 35300470 PMC8862488

[32] Terdiman JP Gruss CB Heidelbaugh JJ Sultan S Falck-Ytter YT. American Gastroenterological Association Institute guideline on the use of thiopurines, methotrexate, and anti-TNF-α biologic drugs for the induction and maintenance of remission in inflammatory Crohn's disease. Gastroenterology. (2013) 145:1459–63. doi: 10.1053/j.gastro.2013.10.047, PMID: 24267474

[33] Connell WR Kamm MA Ritchie JK Lennard-Jones JE. Bone marrow toxicity caused by Azathioprine in inflammatory bowel disease: 27 years of experience. Gut. (1993) 34:1081–5. doi: 10.1136/gut.34.8.1081, PMID: 8174958 PMC1374358

[34] Yewale RV Ramakrishna BS Doraisamy BV Basumani P Venkataraman J Jayaraman K. Long-term safety and effectiveness of Azathioprine in the management of inflammatory bowel disease: a real-world experience. JGH Open. (2023) 7:599–609. doi: 10.1002/jgh3.12955, PMID: 37744710 PMC10517446

[35] Singh A Mahajan R Kedia S Dutta AK Anand A Bernstein CN. Use of thiopurines in inflammatory bowel disease: an update. Intest Res. (2022) 20:11–30. doi: 10.5217/ir.2020.00155, PMID: 33845546 PMC8831775

[36] Ranjan MK Kante B Vuyyuru SK Kumar P Mundhra SK Golla R. Minimal risk of lymphoma and non-melanoma skin cancer despite long-term use of thiopurines in patients with inflammatory bowel disease: a longitudinal cohort analysis from northern India. J Gastroenterol Hepatol. (2022) 37:1544–53. doi: 10.1111/jgh.15880, PMID: 35501287

[37] Relling MV Gardner EE Sandborn WJ Schmiegelow K Pui CH Yee SW. Clinical pharmacogenetics implementation consortium guidelines for thiopurine methyltransferase genotype and thiopurine dosing. Clin Pharmacol Ther. (2011) 89:387–91. doi: 10.1038/clpt.2010.320, PMID: 21270794 PMC3098761

[38] Fraser AG Orchard TR Robinson EM Jewell DP. Long-term risk of malignancym after treatment of inflammatory bowel disease with Azathioprine. Aliment Pharmacol Ther. (2002) 16:1225–32. doi: 10.1046/j.1365-2036.2002.01297.x, PMID: 12144571

[39] Vestergaard P Rejnmark L Mosekilde L. Methotrexate, Azathioprine, cyclosporine, and risk of fracture. Calcif Tissue Int. (2006) 79:69–75. doi: 10.1007/s00223-006-0060-0, PMID: 16927044

[40] Miller LW. Cardiovascular toxicities of immunosuppressive agents. Am J Transplant. (2002) 2:807–18. doi: 10.1034/j.1600-6143.2002.20902.x, PMID: 12392286

[41] Patel AA Swerlick RA McCall CO. Azathioprine in dermatology: the past, the present, and the future. J Am Acad Dermatol. (2006) 55:369–89. doi: 10.1016/j.jaad.2005.07.059, PMID: 16908341

[42] Roberts RL Gearry RB Barclay ML Kennedy MA. IMPDH1 promoter mutations in a patient exhibiting Azathioprine resistance. Pharmacogenomics J. (2007) 7:312–7. doi: 10.1038/sj.tpj.6500421, PMID: 17001353

[43] Lazarević S Đanic M Al-Salami H Mooranian A Mikov M. Gut microbiota metabolism of Azathioprine: a new Hallmark for personalized drug-targeted therapy of chronic inflammatory bowel disease. Front Pharmacol. (2022) 13:879170. doi: 10.3389/fphar.2022.879170, PMID: 35450035 PMC9016117

[44] Yao D Dong M Dai C Wu S. Inflammation and inflammatory cytokine contribute to the initiation and development of ulcerative colitis and its associated Cancer. Inflamm Bowel Dis. (2019) 25:1595–602. doi: 10.1093/ibd/izz149, PMID: 31287863

[45] Cuffari C Dassopoulos T Turnbough L Thompson RE Bayless TM. Thiopurine methyltransferase activity influences clinical response to Azathioprine in inflammatory bowel disease. Clin Gastroenterol Hepatol. (2004) 2:410–7. doi: 10.1016/S1542-3565(04)00127-2, PMID: 15118980

[46] Osterman MT Kundu R Lichtenstein GR Lewis JD. Association of 6-thioguanine nucleotide levels and inflammatory bowel disease activity: a meta-analysis. Gastroenterology. (2006) 130:1047–53. doi: 10.1053/j.gastro.2006.01.046, PMID: 16618398

[47] Relling MV Gardner EE Sandborn WJ Schmiegelow K Pui CH Yee SW. Clinical pharmacogenetics implementation consortium guidelines for thiopurine methyltransferase genotype and thiopurine dosing: 2013 update. Clin Pharmacol Ther. (2013) 93:324–5. doi: 10.1038/clpt.2013.4, PMID: 23422873 PMC3604643

[48] Moriyama T Nishii R Perez-Andreu V Yang W Klussmann FA Zhao X. NUDT15 polymorphisms alter thiopurine metabolism and hematopoietic toxicity. Nat Genet. (2016) 48:367–73. doi: 10.1038/ng.3508, PMID: 26878724 PMC5029084

[49] Sandborn WJ Su C Sands BE D'Haens GR Vermeire S Schreiber S. Tofacitinib as induction and maintenance therapy for ulcerative colitis. N Engl J Med. (2017) 376:1723–36. doi: 10.1056/NEJMoa1606910, PMID: 28467869

[50] Feagan BG Sandborn WJ Gasink C Jacobstein D Lang Y Friedman JR. Ustekinumab as induction and maintenance therapy for Crohn's disease. N Engl J Med. (2016) 375:1946–60. doi: 10.1056/NEJMoa1602773, PMID: 27959607

[51] Sandborn WJ Gasink C Gao LL Blank MA Johanns J Guzzo C. Ustekinumab induction and maintenance therapy in refractory Crohn's disease. N Engl J Med. (2012) 367:1519–28. doi: 10.1056/NEJMoa1203572, PMID: 23075178

[52] Danese S Vermeire S Zhou W Pangan AL Siffledeen J Greenbloom S. Upadacitinib as induction and maintenance therapy for moderately to severely active ulcerative colitis: results from three phase 3, multicenter, double-blind, randomized trials. Lancet. (2022) 399:2113–28. doi: 10.1016/S0140-6736(22)00581-5, PMID: 35644166

[53] Vande Casteele N Gils A Singh S Ohrmund L Hauenstein S Rutgeerts P. Antibody response to infliximab and its impact on pharmacokinetics can be transient. Am J Gastroenterol. (2013) 108:962–71. doi: 10.1038/ajg.2013.12, PMID: 23419382

[54] Panaccione R Ghosh S Middleton S Márquez JR Scott BB Flint L. Combination therapy with infliximab and Azathioprine is superior to monotherapy with either agent in ulcerative colitis. Gastroenterology. (2014) 146:392–400.e3. doi: 10.1053/j.gastro.2013.10.052, PMID: 24512909

[55] Colombel JF Adedokun OJ Gasink C Gao LL Cornillie FJ D'Haens GR. Combination therapy with infliximab and Azathioprine improves infliximab pharmacokinetic features and efficacy: a post hoc analysis. Clin Gastroenterol Hepatol. (2019) 17:1525–1532.e1. doi: 10.1016/j.cgh.2018.09.033, PMID: 30267864

[56] Gilbert B Schrenzel J. Fecal microbiota transplantation: current status and prospects. Rev Med Suisse. (2019) 15:976–83. doi: 10.53738/REVMED.2019.15.650.0976, PMID: 31066530

[57] Urbančič D Pasha F Šmid A Mlinarič-Raščan I. Personalization of thiopurine therapy: current recommendations and future perspectives. Acta Pharma. (2024) 74:355–81. doi: 10.2478/acph-2024-0030, PMID: 39279525

[58] Park JM Han NY Han YM Chung MK Lee HK Ko KH. Predictive proteomic biomarkers for inflammatory bowel disease-associated cancer: where are we now in the era of the next generation proteomics? World J Gastroenterol. (2014) 20:13466–2476. doi: 10.3748/wjg.v20.i37.13466, PMID: 25309077 PMC4188898

[59] Masoodi I Tijjani BM Wani H Hassan NS Khan AB Hussain S. Biomarkers in the management of ulcerative colitis: a brief review. Ger Med Sci. (2011) 9:Doc03. doi: 10.3205/000126 21394194 PMC3046642

[60] Ashton JJ Green Z Kolimarala V Beattie RM. Inflammatory bowel disease: long-term therapeutic challenges. Expert Rev Gastroenterol Hepatol. (2019) 13:1049–63. doi: 10.1080/17474124.2019.1685872, PMID: 31657969

[61] Ma C Solitano V Danese S Jairath V. The future of clinical trials in inflammatory bowel disease. Clin Gastroenterol Hepatol. (2025) 23:635–9. doi: 10.1016/j.cgh.2024.06.036 39025252

[62] Chan PP Wasinger VC Leong RW. Current application of proteomics in biomarker discovery for inflammatory bowel disease. World J Gastrointest Pathophysiol. (2016) 7:27–37. doi: 10.4291/wjgp.v7.i1.27, PMID: 26909226 PMC4753187

